# Influence of Different Feed Physical Forms on Mandibular Gland in Growing Pigs

**DOI:** 10.3390/ani10050910

**Published:** 2020-05-24

**Authors:** Cecilia Dall’Aglio, Francesca Mercati, Elena De Felice, Federico Maria Tardella, Josef Kamphues, Maria Grazia Cappai, Paola Scocco

**Affiliations:** 1 Department of Veterinary Medicine, University of Perugia, Via San Costanzo 4, 06126 Perugia, Italy; cecilia.dallaglio@unipg.it; 2 School of Biosciences and Veterinary Medicine, University of Camerino, Via Pontoni 5, 62032 Camerino, Italy; elena.defelice@unicam.it (E.D.F.); dtfederico.tardella@unicam.it (F.M.T.); paola.scocco@unicam.it (P.S.); 3 Institute of Animal Nutrition, University of Veterinary Medicine Hannover, Foundation, BischofsholerDamm 15, D-30173 Hannover, Germany; josef.kamphues@tiho-hannover.de; 4 Department of Veterinary Medicine, University of Sassari, Via Vienna 2, 07100 Sassari, Italy; mgcappai@uniss.it

**Keywords:** pig, mandibular gland, feed physical form, apelin, aquaporin 5, complex carbohydrates

## Abstract

**Simple Summary:**

The study was carried out on growing pigs fed with different dietary treatments based on different grinding intensities and compactions of the same diet. Chewing acts are associated with salivary production and different extents of saliva fluidity also depend on the specific glycoconjugate content. Therefore, in order to have information about the modifications induced by different feed physical forms in the pig mandibular gland, the glycohistochemical profile and the presence of aquaporin 5, a channel protein modulating the saliva fluidity, were investigated. In addition, to have wider information about the apelinergic system function, presence and localization of both apelin and its receptor were studied. Findings suggest that the different mechanical stimuli in the mouth linked to different feed physical forms likely allow one to diverse physiological behavior of the pig mandibular gland. The intense chewing activity linked to the highest feed compaction and hardness promotes an increase in pig mandibular gland secretion. In addition, saliva becomes more fluid and richer in acid glycoconjugates in order to better lubricate the bolus and protect the mouth mucosae. The apelinergic system is likely involved in the above modifications enhancing both the fluidity and the quantity of serous saliva by the pig mandibular gland.

**Abstract:**

A study was performed on the mandibular gland obtained from growing pigs enrolled in a wide research project aiming to test the effects of different feed physical forms on animal health, production and welfare. We used 48 pigs fed for four weeks with different dietary treatments based on different grinding intensities and compactions of the same diet, namely coarsely ground meal (CM), finely ground pelleted (FP) and coarsely ground pelleted (CP) diets. Samples were analyzed by conventional histochemistry to identify the glycohistochemical profile and by immunohistochemistry to localize aquaporin 5, apelin and apelin receptor. Statistical elaborations were performed using the Stats R-package, version 3.5.3. Pig mandibular gland adenomere increased both the quantity and acidity of produced glycoconjugates from CM to FP and CP diets. This probably calls forth higher watery saliva, thus promoting a better feed softening facilitating the amalgamation of the bolus. Mandibular gland increased aquaporin 5 positivity in the CP diet, supporting the hypothesis of an augmented demand for water. Based on apelin/receptor localization, it was hypothesized that in pig mandibular gland the apelinergic system likely performs an endocrine control on the demilunes activity and a paracrine control on ducts, facilitating the production of a more fluid saliva.

## 1. Introduction

A number of scientific investigations have established that the feed physical form can have an impact on the morphological characteristics and functional activity of the gastrointestinal tracts as well as on associated organs, especially salivary glands of domestic animals [1,2]. In addition, different levels of moisture content in the diet appear involved and may be reasonably expected if liquid or dry diets are provided. In particular, in laboratory animals, a diet with increasing dry matter content is capable of stimulating a progressively more intense chewing activity, with a consistent increase in the absolute fresh weight of mandibular glands (MG). On the contrary, wet to liquid diets were observed to be associated with a reduced organ weight [3,4,5,6]. Such morphological changes are also often associated with qualitative changes in the saliva composition that can be related to different functionalities of the gland, as a result of more or less intense chewing activity [7,8].

More recently, a series of studies involving food-producing animals, especially pigs, were carried out to explore the dietary modulation of salivary gland morpho-functional traits. The findings reported in the literature showed that diets with different moisture contents, thus with different physical characteristics requiring different chewing activity to allow the softening and swallowing of the bolus, are able to determine morphological modifications of the MG in these animals, whereas parotid glands appeared not to be involved [4,5,6,7,8]. Such effects are also associated with variations in the expression of some molecules likely involved in the functional control of the gland itself, such as leptin production and the expression of its receptor, as well as the expression of endocannabinoid receptors [1,2].

Apelin, likewise leptin, is an adipokine produced predominantly, but not exclusively, by the adipose tissue and binding to the specific G-protein-coupled receptors for endogenous ligands. The apelinergic system is a complex system including the peptide apelin (APLN) and its related receptor (APLNR) [9]. APLN and APLNR are extensively expressed in many tissues either in human and different animal species, both laboratory and farm ones [10,11,12,13,14,15]. The literature regarding APLN is scanty and the presence and distribution of the apelinergic system in the salivary glands of the different animal species is not available to date, even though the presence of APLN in human saliva has been recently demonstrated [16]. So far, our work meant to provide additional insights regarding the APLN system in the MG of the pig in view of the effect of different feed physical forms of one same diet. It was, therefore, considered to explore the presence and distribution of the apelinergic system in the MG of growing pigs and its variation as a consequence of more or less intense chewing of the feed, stimulating a consistent effect of the functional activity of the MG. It is well known that chewing acts are associated with salivary production and that different extents of saliva fluidity also depend on the specific glycoconjugate content [17,18]. Therefore, in order to have additional information about the modifications induced in MG functions of the pig in view of different feed physical forms, MG’s glycohistochemical profile was investigated. In fact, complex carbohydrates composing the saliva are able to call forth different amounts of water into the saliva, depending on their chemical functional groups. To support this, we also investigated the presence of aquaporin 5 (AQP5), a water protein channel modulating the water amount in saliva production [19,20,21]. AQP5 pertains to Mammalian aquaporins (AQPs), a class of integral membrane proteins facilitating the rapid and passive moving of water [22]. AQPs have a sequence of about 270 amino acids that form two hemichannels derived by six helical domains spanning the lipid bilayer and resulting in an hourglass-shaped channel for water [23,24,25]. Some of the AQPs are mainly water selective, but there are some AQPs, named aquaglyceroporins, that also transport glycerol, urea and neutral solutes [26,27]. Mandibular gland represents a good organ model being a mixed exocrine gland, whose ademomeres are constituted by a mucous acinar preterminal portion and a serous demilunar portion.

This study is aimed to investigate the influence of different feed physical forms in growing pig’s mandibular gland analyzing the glycohistochemical profile, the presence of AQP5 and localizing the apelinergic system components.

## 2. Materials and Methods

### 2.1. Dietary Treatments and Sampling Collection

The project was approved by the Ethics Committee on Animal Welfare of the Hannover District Government in accordance with the German legislation on animal welfare.

The experiment was conducted using 48 castrated male growing pigs (German Landrace x Large White on Duroc sires) which received a control diet, namely coarsely ground meal diet (CM, dMEAN, 0.88 mm). Raw ingredients were differently processed to obtain pelleted feeds, in particular, finely ground pelleted diet (FP, dMEAN, 0.46 mm); coarsely ground pelleted diet (CP, dMEAN, 0.84 mm) [1]. Feed components and chemical composition are reported in Supplementary Table S1.

The initial total number of animals was divided into three groups of 16 animals and each group received one of the three diets, i.e., CM, FP and CP, from a six-week age for four weeks. [1]. The numerousness of animals for each group was calculated and considered optimal for a significance level of 0.05, a test power of 0.8 and an effect size of 1. Animals were fed ad libitum and had free access to water. At the end of the trial the animals were euthanized according to the European Union regulation on the protection of animals at the time of slaughter (Council Regulation EC No. 1099/2009).

The MG specimens were immediately removed and fixed in buffered formaldehyde (2.5% *v/v*) for 24 h at room temperature and subsequently processed for embedding in paraffin, following routine tissue preparation procedures [28,29].

### 2.2. Immuno- and Glycohistochemical Treatments

The immunohistochemical reactions were visualized on 5 µm sections, collected on poly-L-lysine-coated glass slides. Briefly, sections, dewaxed and brought to water, were microwaved for 15 min in 10 mM citric acid (pH 6.0) for antigen retrieval. All subsequent steps were carried out in a moist chamber at room temperature (RT), to prevent the evaporation of reagents; while, to prevent non-specific binding of primary antibody and before using the primary antibody, the sections properly cooled were preincubated with the normal serum for 30 min. Subsequently, once the excess of reagent has been removed from the sections, they were incubated, overnight at RT, each with one of the primary antibodies: rabbit polyclonal antibody anti-APLN (1:100, NBP2-31176, Novus Biologicals, Littleton, CO, USA), mouse monoclonal antibody anti-APLNR (1:100, sc-517300, Santa Cruz Biotechnology, Santa Cruz, CA, USA), and rabbit polyclonal antibody anti-AQP5 (1:100, AQP-005, Alomone Labs, Jerusalem 9104201, Israel). The specificity of each primary antibody used, verified by blasting the full protein sequences with corresponding swine ones, is shown in Table S2.

The next day, after washing in PBS, the sections were incubated at RT for 30 min with the corresponding secondary antibody and subsequently processed for 30 min using the avidin–biotin complex (ABC KIT, PK-6100, Vector Laboratories, Burlingame, CA, USA) and the DAB (SK-4100, Vector Laboratories, Burlingame, CA, USA) as the chromogen. The corresponding secondary biotinylated antibodies were: horse anti-rabbit (1:200, BP-1100, Vector Laboratories, Burlingame, CA, USA) and horse anti-mouse IgG antibodies (1:200, BP-2000, Vector Laboratories, Burlingame, CA, USA). At the end of the immunoreaction, the sections were rinsed in PBS, counterstained or not with hematoxylin, dehydrated and mounted in Eukitt.

As positive controls sheep abomasum was used for both APLN and APLNR [14], while sheep MG was used for AQP5 [30]. Sections in which the primary antibodies were omitted or substituted with preimmune gamma globulin were used as controls of non-specific staining.

Carbohydrate characterization was performed on 5 µm sections by staining with periodic acid–Schiff (PAS, evidencing vicinal diols), Alcian blue (AB) pH 2.5 (evidencing acid groups), AB/PAS, AB pH 1 (evidencing sulfated groups), AB pH 0.5 (evidencing highly sulfated groups) [31].

All tissue analyses were carried out on coded slides using a light microscope (Nikon Eclipse E800, Nikon Corporation, Tokyo, Japan) connected to a digital camera (Dxm 1200 Nikon digital camera). Images were processed using an image analysis system (Lucia, Laboratory Imaging Ltd., Prague, Czech Republic). The settings for image capture were standardized by subtracting the background signals obtained from the marched tissue sections which had not reacted with the primary antibodies and which were used as immunohistochemical controls.

For each animal, three independent observers, unaware of the treatments carried out, evaluated five microscopic fields of each experimental group and the intensity of the staining was graded in arbitrary units as follows: negative (–), weak (±), moderate (+), strong (++) and very strong (+++).

Variations in the intensity of immunopositivity for APLN, APLNR and AQP5 were observed among different groups of animals, probably reflecting the expression of the corresponding antigens. Even if the immunohistochemical technique shows a prevalently qualitative nature [32], a semiquantitative evaluation of immunopositivity was performed using the same scale applied to conventional glycohistochemistry.

### 2.3. Statistical Analyses

To test the null hypothesis of no difference among different experimental groups for each immune- and glycohistochemical treatment, we performed one-way ANOVAs if the variables satisfied conditions for parametric tests (normality was tested using the Shapiro–Wilk test); homogeneity of variance was ANOVA/Kruskal–Wallis test. We ran pairwise comparisons using the independent samples *t*-test or Wilcoxon–Mann–Whitney test to identify which groups were significantly different from each other. To test the null hypothesis that the location shift between groups is equal to 0, we performed two Wilcoxon signed-rank tests between the serial treatments AB pH 2.5 vs. AB pH 1 and AB pH 1 vs. AB pH 0.5. In both the analyses, a Holm correction for multiple comparisons was used to avoid type I error.

Statistical elaborations were performed using the R version 3.5.3 (R Core Team 2019, Vienna, Austria), the stats R-package, version 3.5.3 (shapiro.test, aov, kruskal.test, t.test, wilcox.test, Vienna, Austria), and the *car* R-package, version 3.0-2 (leveneTest function, Vienna, Austria) [33].

## 3. Results

### 3.1. Immunohistochemistry

**Figure 1:**
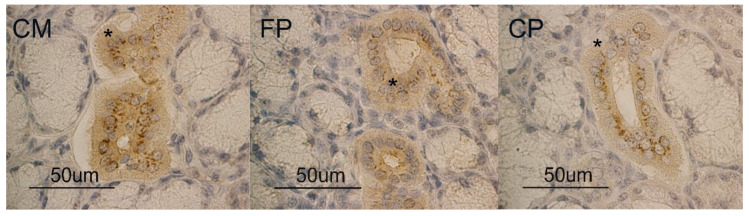
Pig mandibular gland. Apelin (APLN) binding sites at duct (*) level in coarsely ground meal (CM), finely ground pelleted (FP) and coarsely ground pelleted (CP) groups.

The immunohistochemistry showed APLN binding sites at the duct cell level in the pig mandibular gland for the three diet groups. In particular, the moderate APLN reactivity observed in CM diet (Figure 1 CM) was slightly decreased in both FP and CP diets showing a similar reactivity (Figure 1 FP, CP).

**Figure 2:**
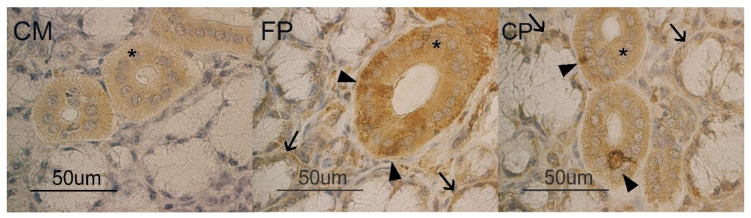
Pig mandibular gland. APLNR binding sites at duct (*) and demilune (**↑**) level in CM, FP and CP groups. In FP and CP samples, some cells (arrowhead) have a higher reactivity than others.

With regards to APLNR reactivity in the pig mandibular gland, in the CM diet, only a weak positivity was observed in ductal cells (Figure 2 CM). In FP and CP diets, a moderate reactivity to APLNR appeared in the mandibular gland demilunes and ducts. In addition, in both FP and CP samples, at duct level, it was possible to observe a few cells strongly APLNR reactive (Figure 2 FP, CP). In addition, the morphological observation of the different diet samples seemed to suggest an increase in demilune size.

**Figure 3:**
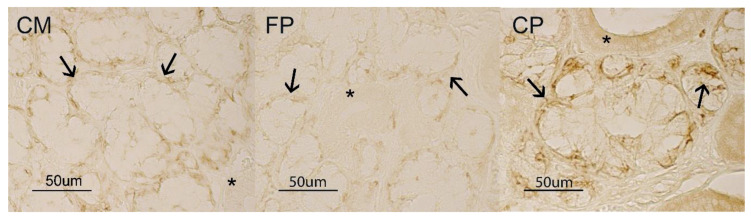
Pig mandibular gland. AQP5 binding sites at duct (*) and demilune (↑) level in CM, FP and CP groups.

As for the other immunohistochemical treatments, pig mandibular acini did not react to AQP5 antibody. On the contrary, demilunes showed a weak positivity in CM and FP samples, which became strong in the CP diet samples. Additionally, in the CP diet, a slight AQP5 positivity was seen in the ducts, above all at cell coat level (Figure 3).

Sample reactivities to immunohistochemical treatments are summarized in Table 1.

Sheep abomasum and MG, used as positive controls, showed a binding pattern for APLN, APLNR and AQP5 (Figure S1), while staining was completely absent in the control sections where the primary antibodies were omitted and in sections incubated with normal rabbit IgG (Figure S2).

### 3.2. Glycohistochemistry

**Figure 4:**
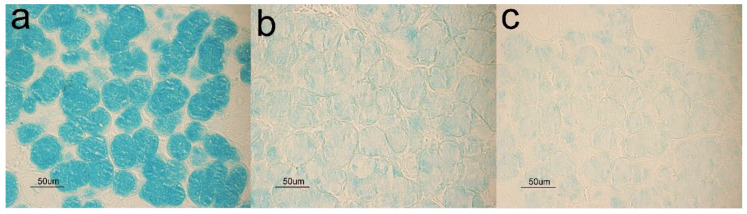
Pig mandibular gland. CP group shows mucous acini strongly reactive to Alcian Blue (AB) pH 2.5 (**a**), moderately positive to AB pH 1 (**b**) and weakly stained with AB pH 0.5 (**c**).

Glycohistochemical treatments evidenced a higher production of complex carbohydrates by the pig mandibular acini in FP and CP diets with respect to CM. In addition, comparing the reactivity of AB pH 2.5, AB pH1 and AB pH 0.5 among the three diets showed an increased production of acid glycoconjugates, particularly in the CP diet where acinar cells showed a weak positivity also to AB pH 0.5 (Figure 4).

Sample reactivities to glycohistochemical treatments are summarized in Table 2.

### 3.3. Statistical Analysis

The significance of differences among the diet groups for each immuno- and glycohistochemical treatment are shown in Table 3.

The significance of the differences among the serial treatments AB pH 2.5, AB pH 1 and AB pH 0.5 for the three diet groups are shown in Table 4.

Glycohistochemical evidence allows one to hypothesize that secretory structures of pig MG produce the complex carbohydrate types listed, in descending semi-quantitative order, in Table 5.

## 4. Discussion

The glycohistochemical and immunohistochemical investigations were carried out on the MG obtained from pigs fed with different dietary treatments based on different grinding intensities and compactions of the same diet [1]. Animals were enrolled in a wide research project, running in the Institute of Animal Nutrition of the University of Hannover, aiming to test the effects of different feed of physical forms on the health, production and welfare of pigs. This research extends the knowledge about the diet modifications on pig welfare [31,34] and could be useful for further investigation also related to diet and farm income relationships [35].

Our findings allow considering that the different feed physical form is capable of inducing morphological and functional modification of the MG, on the basis of presence and distribution of molecules differently modulating the saliva composition.

The different compaction of the diet can reasonably produce different perception extents of the physical form of the bolus, with consequent different voluntary chewing and adaptation of salivary production. In response to the feed physical form, demilunes of pig MG increase in size and the acidity of produced glycoproteins change in view of CM vs. FP vs. CP diets. Acinar cells increase their production of hyaluronic acid-/chondroitin-like Glycosaminoglycans (GAGs) in FP and CP groups; in addition, in the MG of pigs fed with the CP diet a low amount of heparin/heparan-sulfate-like GAGs production is stimulated. Additional production of complex carbohydrates observed in MG from pigs of FP and CP groups is able to call forth higher watery saliva, thus promoting a better feed softening facilitating the formation and amalgamation of the bolus. A handful of evidence pointed out that the increased glycoconjugate production, especially when they are highly acid, allows drawing a large amount of water in many organ tissues and in different animal species [36,37,38,39,40,41]. In addition, complex carbohydrates could envelope pathogenic bacteria, viruses and parasites acting as hapten-like binding sites, preventing their attachment to the mucosae potentially damaged by the harder feed [34,42,43].

Pig MG demilunes are weakly reactive to AQP5 in CM and FP diet but increase their positivity in the CP diet samples. The latter also shows AQP5 binding sites in the ducts. This evidence further supports the hypothesis of an augmented demand for water in the mouth. It was stated that AQP5 in the salivary glands is probably involved in providing for a suitable amount of fluid to be secreted [20,44]; in addition, its expression can be affected by feed and environmental modification, in particular at serous adenomere level [30].

APLN binding sites observed in ducts of MG from the pigs fed with the CM diet decrease their reactivity in MG of samples from FP and CP dietary groups. On the contrary, such structures increased their reactivity to APLNR from CM to FP and CP samples. In addition, in the two last groups, there was a strong positivity for APLNR in some cells. Demilunes also showed binding sites for APLNR in FP and CP diet samples. Comparing the specific APLN and APLNR localization and the differences in the binding sites reactivity among the CM, FP and CP diet samples, it is likely to hypothesize that in pig MG the apelinergic system performs an endocrine control on the demilunes activity facilitating the production of a more fluid saliva. In addition, pig MG ducts seem to be affected by a paracrine control by the apelinergic system, which could allow an inhibition in the water absorption, enhancing the saliva water content.

According to the above considerations, the literature states that adipokines can act through endocrine action as well as paracrine and autocrine ones. The interaction of those mechanisms has already been described in a wide range of physiological and physio-pathological processes in different animal species [45,46,47]. A paracrine action for apelin was already suggested for sheep uterus [15] and mammary gland [14] where the molecule aimed at regulating the gland secretive action. Up to now the apelin action on salivary glands has not yet been described. However, the role of this adipokine on the gastroenteric apparatus is well known and numerous studies suggest a role for apelin in both exocrine and endocrine functions [48,49,50]. Apelin performs important functions in the pancreas, an organ that shows, as its exocrine parenchyma regards, similar anatomical structures and physiological functions with salivary glands [51]. Particularly, apelin injected intravenously decreased pancreatic juice volume, protein and trypsin outputs in a dose-dependent manner while intraduodenal administration of apelin increases pancreatic protein and trypsin secretion [52].

Finally, in pig mandibular gland it was stated that different feed physical forms affect the expression of both the leptinergic system and cannabinoid type 1 and 2 receptors [1,2], as now observed for the apelinergic system.

## 5. Conclusions

In conclusion, obtained data suggest that the diverse feed physical forms, performing differentiated mechanical stimuli in the mouth, likely allow a different physiological behavior of pig MG. The intense chewing activity linked to the highest feed compaction and hardness promotes an increase in pig MG secretion; in addition, saliva becomes more fluid and richer in acid glycoconjugates in order to better lubricate the bolus and protect the mouth mucosae. The apelinergic system is likely involved in the above modifications enhancing both the fluidity and the quantity of serous saliva by the pig mandibular gland.

## Figures and Tables

**Table 1:** Sample reaction intensity expressed in arbitrary units toward immunohistochemical targets.

Antigen	Secretory Structures	Diet Groups		
		CM	FP	CP
APLN	Acini	−	−	−
	Demilunes	−	−	−
	Ducts	+	±/+	±/+
APLNR	Acini	−	−	−
	Demilunes	−	+	+
	Ducts ^a^	±	+	+
	Ducts ^b^	−	++	++
APQ5	Acini	−	−	−
	Demilunes	±	±	+
	Ducts	−	−	±

CM: coarse meal diet; FP: fine pellet diet; CP: coarse pellet diet. ^a^ Epithelial lining; ^b^ epithelial more reactive cells. APLN = Apelin; APLNR = Apelin Receptor; AQP5 = Aquaporin 5. Intensity of the staining: – = negative, ± = weak, + = moderate, ++ = strong.

**Table 2:** Sample reaction intensity expressed in arbitrary units toward glycohistochemical treatments.

Glycohistochemical Treatments	Secretory Structures	Diet Groups		
		CM	FP	CP
AB pH2.5	Acini	+/++	++	++
	Demilunes	−/±	±/+	±
	Ducts	−	−	−
AB pH1	Acini	±/+	±/+	±/+
	Demilunes	−	−	−
	Ducts	−	−	−
AB pH0.5	Acini	−	−	−/±
	Demilunes	−	−	−
	Ducts	−	−	−
PAS	Acini	++	++	+++
	Demilunes	±	±	±
	Ducts	−	−	−
AB/PAS	Acini	B+/R++	B++/R++	B+++/R++
	Demilunes	B+/R±	B±/R±	B+/R±
	Ducts	−	−	−

CM: coarse meal diet; FP: fine pellet diet; CP: coarse pellet diet. B = blue; R = red. AB = Alcian Blue; PAS = Periodic Acid Schiff.

**Table 3:** Statistical significance of differences (*p* ≤ 0.01) for each histochemical treatment among different experimental treatments, as performed by one-way ANOVA and Kruskal–Wallis tests and respective pairwise comparisons, as performed by independent samples *t*-test and Wilcoxon–Mann–Whitney tests. *P-*values were adjusted for multiple testing using the Holm correction.

Histochemical Treatments		ANOVA/Kruskal–Wallis Test	*t*-test/Wilcoxon–Mann–Whitney Test		
			*P* CM vs. FP	*P* FP vs. CP	*P* CM vs. CP
APLN	Acini	−	−	−	−
	Demilunes	−	−	−	−
	Ducts	4.26 × 10^−11^	1.76 × 10^−7^	1.00	1.76 × 10^−7^
APLNR	Acini	−	−	−	−
	Demilunes	1.92 × 10^−6^	5.50 × 10^−6^	1.00	5.50 × 10^−6^
	Ducts ^a^	<10^−^^16^	8.36 × 10^−13^	1.00	1.61 × 10^−12^
	Ducts ^b^	1.60 × 10^−6^	5.50 × 10^−6^	1.00	5.50 × 10^−6^
APQ5	Acini	−	−	−	−
	Demilunes	3.16 × 10^−6^	1.00	1.92 × 10^−5^	1.92 × 10^−5^
	Ducts	6.57 × 10^−^^9^	−	5.50 × 10^−6^	5.50 × 10^−6^
AB pH2.5	Acini	3.16 × 10^−6^	1.92 × 10^−5^	1.00	1.92 × 10^−5^
	Demilunes	2.33 × 10^−8^	1.80 × 10^−5^	1.90 × 10^−5^	1.92 × 10^−5^
	Ducts	−	−	−	−
AB pH1	Acini	1.00	−	−	−
	Demilunes	−	−	−	−
	Ducts	−	−	−	−
AB pH0.5	Acini	6.12 × 10^−^^9^	−	5.28 × 10^−6^	5.28 × 10^−6^
	Demilunes	−	−	−	−
	Ducts	−	−	−	−
PAS	Acini	<10^−^^16^	1.00	<10^−^^16^	<10^−16^
	Demilunes	1.00	−	−	−
	Ducts	−	−	−	−
AB/AS	Acini	<10^−^^16^	1.08 × 10^−13^	4.08 × 10^−14^	<10^−16^
	Demilunes	1.03 × 10^−^^12^	1.24 × 10^−9^	1.05 × 10^−10^	1.00
	Ducts	−	−	−	−

CM: coarse meal diet; FP: fine pellet diet; CP: coarse pellet diet. ^a^ Epithelial lining; ^b^ epithelial more reactive cells. APLN = Apelin; APLNR = Apelin Receptor; AQP5 = Aquaporin 5; AB = Alcian Blue; PAS = Periodic Acid Schiff.

**Table 4:** Statistical significance of differences (*p* ≤ 0.01) among different pH AB serial treatments as performed by Wilcoxon signed-rank tests. *P-*values were adjusted for multiple testing using the Holm correction.

Diet Groups	Secretory Structures	Wilcoxon Signed-Rank Test	
		AB pH 2.5 vs. AB pH 1	AB pH 1 vs. AB pH 0.5
CM	Acini	0.0035	0.0035
	Demilunes	0.0035	−
	Ducts	−	−
FP	Acini	0.0035	0.0035
	Demilunes	0.0035	−
	Ducts	−	−
CP	Acini	0.0035	0.0035
	Demilunes	0.0035	−
	Ducts	−	−

CM: coarse meal diet; FP: fine pellet diet; CP: coarse pellet diet. AB = Alcian Blue

**Table 5:** Kinds of glycoconjugates produced by the pig mandibular gland (MG) secretory structures, listed in descending order as also evidenced by a differentiated style.

Secretory Structures	CM	FP	CP
Acini	Hyaluronic acid and/or Chondroitin-like GAGs Chondroitin sulfate A/B/C-like GAGs	**Hyaluronic acid and/or Chondroitin-like GAGs** Chondroitin sulfate A/B/C-like GAGs	**Hyaluronic acid and/or Chondroitin-like GAGs** Chondroitin sulfate A/B/C-like GAGs Heparin and/or heparan-sulfate-like GAGs
Demilunes	Neutral and acid glycoproteins	Neutral and acid glycoproteins	Acid glycoproteins

CM: coarse meal diet; FP: fine pellet diet; CP: coarse pellet diet. GAGs = Glycosaminoglycans.

## Supplementary Materials

The following are available online at https://www.mdpi.com/2076-2615/10/5/910/s1, Table S1: Feed components and chemical composition. Table S2. Specificity of the primary antibodies used. Figure S1: Immunohistochemistry positive controls. Figure S2: Immunohistochemistry negative controls.
