# Supplementary material for: Influence of Different Feed Physical Forms on Mandibular Gland in Growing Pigs
Source: Animals (Basel). 2020 May 24;10(5):910. doi: 10.3390/ani10050910 (PMC7278392; doi:10.3390/ani10050910)
Supplement: Supplementary file 1 [file animals-10-00910-s001.pdf]

Influence of Different Feed Physical Forms on Mandibular Gland in Growing Pigs

Cecilia Dall'Aglio, Francesca Mercati, Elena De Felice, Federico Maria Tardella, Josef Kamphues, Maria Grazia Cappai, Paola Scocco

Table S1. Feed components and chemical composition.

Component	Percentage
Barley	34
Wheat	40
Soybean meal	20
Soybean oil	1.1
Mineral vitamin Premix	2.2
Skim milk powder	2
L-lysine	0.5
α-Methionine	0.2
**Chemical composition**	**g/Kg of dry material**
Crude ash	45.8
Crude protein	186
Crude fat	37
Crude fiber	42.1
Starch	406

Table S2. Specificity of the primary antibodies used

Antibody used	*Species*	*% identity with swine full protein sequence*
Apelin	human	83.12
Apelin receptor	human	92.69
Aquaporin 5	rat	92.08

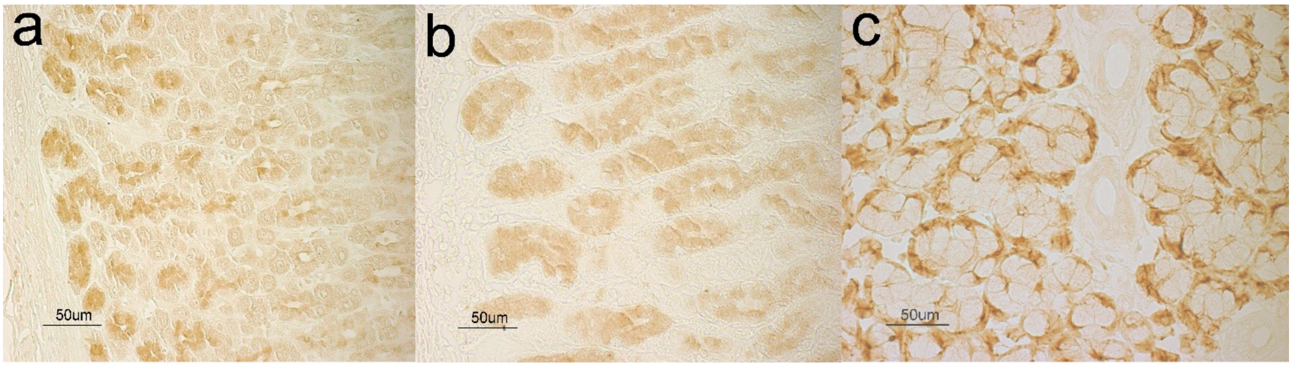

Figure S1. Immunohistochemistry positive controls. Sheep abomasum sample for APLN (**a**) ad APLNR (**b**); sheep mandibular gland for AQP5 (**c**).

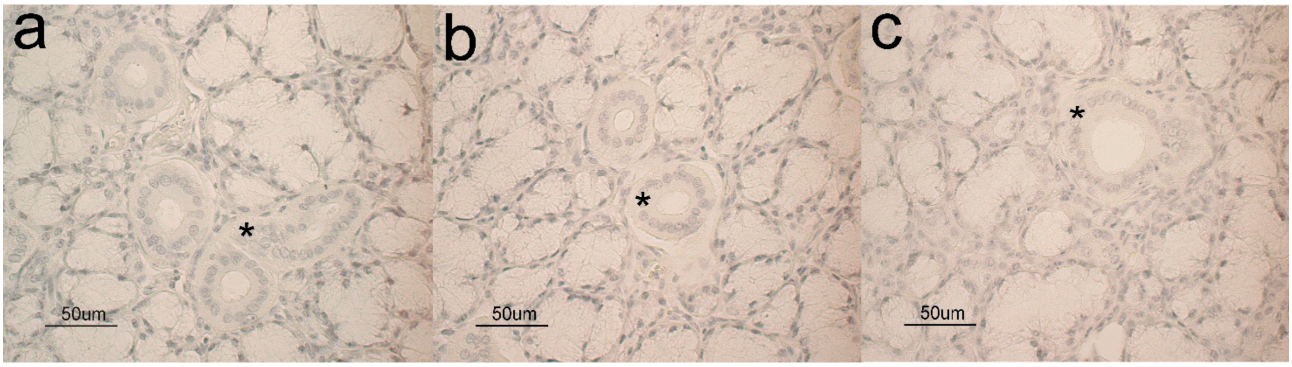

Figure S2. Pig mandibular gland. Immunohistochemistry negative controls for APLN (**a**), APLNR (**b**) and AQP5 (**c**) are treated with hematoxylin to contrast the parenchymal structures. Asterisks (*) indicate the MG ducts.
